# Non-neoplastic Lesions among Lateral Neck Mass Specimens in a Tertiary Care Center: A Descriptive Cross-sectional Study

**DOI:** 10.31729/jnma.6430

**Published:** 2021-07-31

**Authors:** Rachana Dhakal, Ramesh Makaju, Monika Pokharel, Dipika Basnet, Mukta Singh Bhandari

**Affiliations:** 1 Department of Pathology, Dhulikhel Hospital, Kathmandu University School of Medical Sciences, Dhulikhel, Kavre, Nepal; 2 Department of Otorhinolaryngology, Dhulikhel Hospital, Kathmandu University School of Medical Sciences, Dhulikhel, Kavre, Nepal; 3 Department of Community Medicine, Kathmandu University School of Medical Sciences, Dhulikhel, Kavre, Nepal

**Keywords:** *cytology*, *lymph node*, *thyroid gland*

## Abstract

**Introduction:**

Lateral neck masses present clinically as neoplastic or non-neoplastic lesions of lymph nodes, salivary glands, and thyroid. Non-neoplastic lesions, if evaluated timely, may not transform into malignancy thus reducing clinical burden. A cytomorphological study using fine needle aspiration is a reliable method for the diagnosis of such masses. The aim of this study was to find out the prevalence of non-neoplastic lesions of lateral neck mass specimens received in the Department of Pathology in a tertiary care center.

**Methods:**

This descriptive cross-sectional study was carried out in the Department of Pathology among lateral neck mass specimens of a tertiary care center from January 2019 to December 2020 after obtaining ethical approval from the Institutional Review Committee (Reference no: 155/19). A convenience sampling method was used and data analysis was done in Microsoft Excel 2019. Point estimate at 95% Confidence Interval was calculated along with frequency and proportion for binary data.

**Results:**

Out of 300 lateral neck mass specimens, non-neoplastic lesions were found in 246 (82%) (77.7-86.3 at 95% Confidence Interval). The involvement of lymph nodes in 117 (47.6%) was the most common finding followed by thyroid 112 (45.5%). Among non-neoplastic lesions, the cytomorphological features of benign nodular goiter 93 (37.8%) was the most common lesion followed by reactive lymphoid hyperplasia 73 (29.7%).

**Conclusions:**

The study showed that the prevalence of non-neoplastic lesions was similar to that of other national and international studies.

## INTRODUCTION

Lateral neck mass is one of the common clinical problems which have a wide pathologic spectrum.^1^ It can be congenital or acquired, neoplastic, or nonneoplastic. These can frequently present as enlarged lymph nodes which can be due to metastatic or inflammatory causes, thyroid disorders, or salivary gland tumors. Other causes could be branchial cyst, thyroglossal cyst, epidermal cyst, or soft tissue tumor.^2^

The clinical evaluation of these lateral neck masses is difficult because of the diversity of lesions. Hence, earlier evaluation of lateral neck masses with cytomorphological study avoids unnecessary surgeries in non-neoplastic or inflammatory conditions.

The objective of this study was to find out the prevalence of non-neoplastic lesions among lateral neck mass specimens received in the Department of Pathology, Dhulikhel Hospital-Kathmandu University Hospital (DH-KUH).

## METHODS

A descriptive cross-sectional study was conducted in the Department of Pathology in a tertiary care center from January 2019 to December 2020. Ethical approval was taken from the Institutional Review Committee of DH-KUH (Reference no: 155/19). In this study, all the patients attending the Department of Pathology, DH-KUH in the study period having fine needle aspiration cytology (FNAC) of lateral neck masses were included. Convenience sampling was done and the sample size was calculated as,

n = Z^2^ × p × (1 - p) / e^2^

  = (1.96)^2^ × (0.5) × (1-0.5) / (0.1)^2^

  = 96

Where,

n = minimum required sample size
Z = 1.96 at 95% Confidence Interval (CI)
p = prevalence, 50%, for maximum sample size
q = 1-p
e = margin of error, 10%

Taking a 10% non-response rate, the sample size was 106. After clinical examination, informed consent was obtained from patients. Under aseptic precautions, FNAC was done using a 22-24 gauge needle fitted to a 10ml disposable syringe. After immobilizing the target swelling, multiple passes were given to get sufficient material. Smears were prepared and stained with May Grunwald Giemsa (MGG) and Papanicolaou (PAP) stains. The Ziehl Neelsen (ZN) stain for Acid Fast Bacilli (AFB) was done in those cases, where clinically suspected as tuberculosis (TB), and also in those cases where purulent or cheesy material was aspirated.

The cytological features evaluated included cellularity (scanty, moderate, and high), cell arrangement, nuclear and cytoplasmic characteristics, and background elements. The cases were analyzed by the primary investigator. Inadequate samples were not included in the study.

The data were collected using proforma consisting of demographic characteristic and cytomorphological variables. The stained slides were examined under a light microscope by the primary investigator. The data were analyzed using Microsoft Excel 2019. Point estimate at 95% Confidence Interval was calculated along with frequency and proportion for binary data.

## RESULTS

Out of 300 specimens collected in the study, the prevalence of non-neoplastic lesions was 246 (82%) (77.7-86.3 at 95% Confidence Interval).

The age of the patients ranged from 3 to 79 years including both male and female, the median age of occurrence being 39 years. Majority of the cases 37 (31.6%) among lymph nodes were in the age group 2029 years, 33 (29.5%) of the thyroid gland lesions were in the age group 50-59 years and 4 (23.5%) of the cases of salivary gland lesions were in the age group 10-19 years. Out of all cases, 11 (4.4%) patients were seen in the age group below 10 years (Table 1).

**Table 1:** Age and site wise distribution of Non-neoplastic lesions of lateral neck masses.

Site	0-9 years n(%)	10-19 years n(%)	20-29 years n(%)	30-39 years n(%)	40-49 years n(%)	50-59 years n(%)	60-69 years n(%)	70-79 years n(%)	Total n(%)
Lymph node	9 (3.7)	15 (6.1)	37 (15.0)	15 (6.1)	14 (5.7)	11 (4.5)	10 (4.1)	6 (2.4)	117 (47.6)
Thyroid	1 (0.4)	2 (0.8)	14 (5.7)	14 (5.7)	23 (9.3)	33 (13.4)	18 (7.3)	7 (2.8)	112 (45.5)
Salivary gland	1 (0.4)	4 (1.6)	3 (1.2)	3 (1.2)	2 (0.8)	2 (0.8)	1 (0.4)	1 (0.4)	17 (6.9)
Total	11 (4.5)	21 (8.5)	54 (21.9)	32 (13.0)	39 (15.9)	46 (18.7)	29 (11.8)	14 (5.7)	246 (100)

There were 87 (35.3%) male and 159 (64.7%) female patients. The incidence of lymph node lesion was significantly higher in females 62 (53%) than males 55 (47%). Similarly, in thyroid lesions, 89 (79.5%) were females and 23 (20.5%) were males. The incidence of salivary gland lesion was slightly higher in male 9 (53%) (Table 2).

**Table 2:** Sex and site wise distribution of non-neoplastic lesions of lateral neck masses.

Site	Male n (%)	Female n (%)	Total n (%)
Lymph node	55 (47)	62 (53)	117 (100)
Thyroid	23 (20.5)	89 (79.5)	112 (100)
Salivary gland	9 (53)	8 (47)	17 (100)
Total	87 (35.3)	159 (64.7)	246 (100)

The distribution of cytomorphological spectrum of nonneoplastic lesions of lateral neck masses result showed lymph nodes lesion 117 (47.6%) as the predominant site of FNAC followed by thyroid lesions 112 (45.5%) and salivary gland lesions 17 (6.9%). Among 117 cases of lymph node lesions, reactive lymphoid hyperplasia 73 (29.7%) was the main cause of lymphadenopathy followed by tubercular lymphadenitis 29 (11.8%) and nonspecific/suppurative inflammatory lesions 15 (6.1%). Out of 112 thyroid lesions, benign nodular goiter lesions were 93 (37.8%) and lymphocytic thyroiditis (including Hashimoto's thyroiditis) 19 (7.7%). Among 17 (6.9%) salivary gland lesions, all the lesions were diagnosed as chronic sialadenitis (Table 3).

**Table 3:** Spectrum of non-neoplastic lesions of lymph node, thyroid and salivary gland masses.

Site	Lesion	No. of cases n (%)
Lymph node	Reactive lymphoid hyperplasia	73 (29.7)
	Tubercular Lymphadenitis	29 (11.8)
	Nonspecific /Suppurative	15 (6.1)
Thyroid	Benign Nodular Goiter (Colloid Goiter)	93 (37.8)
	Lymphocytic thyroiditis (including Hashimoto's thyroiditis)	19 (7.7)
Salivary Gland	Chronic sialadenitis	17 (6.9)
Total		246 (100)

## DISCUSSION

Lateral neck mass is one of the challenging diagnostic problems to the clinician. Although surgical biopsy is the gold standard method of tissue diagnosis, FNAC has become one of the first line investigation method to evaluate the lateral neck masses.^1^ A lateral neck mass may suggest a wide range of entities in a differential diagnosis, including reactive, infectious, developmental, and neoplastic conditions.^3^

In the present study, cytomorphological features of 246 cases of non-neoplastic neck masses were analyzed. Maximum numbers of cases were observed in the age group of 20 to 29 years and 50 to 59 years respectively. Our study is similar to the study by Agrawal, et al. which showed female preponderance and similar age group distribution.^4^ Similarly, Soni, et al. observed the involvement of 66.15% female and 33.89% males which were similar to our study which constituted 35.3% male and 64.7% female.^5^

Reactive lymphadenopathy may present as a tender, mobile swelling and should be considered in the context of a recent infection or regional injury. Resolution of the underlying infectious or inflammatory origin results in normalization of the node within weeks.^6^ In the various pathological spectrum of lymphadenopathies, reactive hyperplasia constituted the majority of the cases (29.7%). The findings were similar to the study conducted by Agrawal et al. which constituted lymphoid hyperplasia as the predominant cause of lymphadenopathy.^4^

In the present study, tubercular lymphadenitis (11.8%) was the other common lesion among lymph nodes.

Though the diagnosis of tuberculosis can be made by Enzyme Linked Immunosorbent Assay (ELISA) and Polymerase Chain Reaction (PCR) but these methods are very costly and are not available at many centers.^7^ Hence, FNA smears with ZN stain is the first line diagnostic technique.^8^ With the prompt diagnosis and treatment of tuberculosis morbidity and mortality can be reduced.^9^

Thyroid lesions are the commonly encountered lesion in the world and FNAC is a safe and cost-effective diagnostic modality in screening thyroid masses. In our study, among thyroid lesions, colloid goiter was the commonest lesion which constituted about 37.8% of cases. Rout, et al. also observed colloid goiter as the common thyroid swelling (42.2%) in his study.^10^

In thyroid aspiration cytology, marked cellular smear is one of the common issues, where increased cellularity with loss of cohesion may be present in hyperplastic /adenomatous goiter, adenoma, or carcinoma. The importance of earlier diagnosis of thyroid lesion by using FNAC is that thyroiditis and colloid goiter can be managed with a conventional method, likewise, lymphomas and undifferentiated carcinomas may be managed with radiotherapy or chemotherapy.^11^ Hence, earlier diagnosis of such lesions through aspiration cytology prevents surgical intervention.

Chronic sialadenitis is one of the pitfalls in salivary gland cytopathology as the associated malignancy might not be aspirated which leads to a false-negative diagnosis. Besides, the presence of reactive cellular atypia might also lead to over diagnosis and patient might undergo unnecessary surgery.^12,13^ In this study, all the salivary gland lesions were diagnosed as chronic sialadenitis which accounted for 6.9% which was similar to findings from study by Manjula, et al.^14^

## CONCLUSIONS

The prevalence of non-neoplastic lesion was high among the lateral neck masses with about four in five specimens being non-neoplastic. Benign nodular goiter was the most common cytomorphological findings in the thyroid gland. Reactive lymphoid hyperplasia and chronic sialadenitis were the main lesions among lymph nodes and salivary gland respectively.
